# Extracting novel information from neuroimaging data using neural fields

**DOI:** 10.1186/1471-2202-15-S1-O4

**Published:** 2014-07-21

**Authors:** Dimitris A Pinotsis

**Affiliations:** 1 The Wellcome Trust Centre for Neuroimaging, University College London, WC1N 3BG, UK

## 

This talk will introduce new links between the theory of differential equations and the analysis of neuroimaging data. We will focus on a class of population models called neural fields: these are models of how the brain is wired [1] and how it responds in different experimental conditions, which embody topographic features of cortical sources [2]. We will demonstrate how neural fields can be used to interpret brain responses measured with electrophysiology [3]. The inversion of such models is based upon Bayesian techniques and provides estimates of biologically and functionally meaningful quantities among different experimental conditions.

Neural fields model current fluxes as continuous processes on the cortical sheet, using partial differential equations (PDEs). The key advance that neural field models offer, over other population models (like neural masses), is that they embody spatial parameters (like the density and extent of lateral connections). This allows one to model responses not just in time but also over space. Conversely, these models are particularly useful for explaining observed cortical responses over different spatial scales; for example, with high-density recordings, at the epidural or intracortical level. However, the impact of spatially extensive dynamics is not restricted to expression over space but can also have profound effects on temporal (e.g., spectral) responses at one point (or averaged locally over the cortical surface)[4]. This means that neural field models may also play a key role in the modelling of non-invasive electrophysiological data that does not resolve spatial activity directly.

We will shed light on different uses of neural fields and put forward three reasons why these models can be useful in the analysis of neuroimaging data. Each of these motivations is demonstrated by analysing a particular dataset obtained using three different modalities: electrocorticography (ECoG), magnetoencephalography (MEG) and local field potential recordings (LFPs). We will argue that neural fields allow one to: (i) compare evidences for alternative hypotheses regarding the important neurobiological determinants of stimulus-specific response variability[5]; (ii) make inferences about between subject variability in cortical function and microstructure using non-invasive data [6] and (iii) obtain estimates of spatial parameters describing cortical sources in the absence of spatially resolved data [7].

Our analyses exploit dynamic causal modelling [8] and include model space explorations that embody different hypotheses about the generation of observed responses in relation to model evidence - obtained using Variational Bayes [9]. This model comparison uses a variational free-energy bound to furnish optimized models in a manner similar to fitting empirical spectra with AR and ARMA models, see e.g.[10]. The advantage this approach has over other optimization criteria is that it provides an optimal balance between model fit and complexity; yielding models that are both parsimonious and accurate. The analyses presented here showcase particular instances where neural field models serve as a mathematical microscope, allowing us to extract information that is hidden in electrophysiological data.
